# A Methodology for Discriminant Time Series Analysis Applied to Microclimate Monitoring of Fresco Paintings

**DOI:** 10.3390/s21020436

**Published:** 2021-01-09

**Authors:** Sandra Ramírez, Manuel Zarzo, Angel Perles, Fernando-Juan García-Diego

**Affiliations:** 1 Department of Applied Statistics, Operations Research and Quality, Universitat Politècnica de València, Camino de Vera, s/n 46022 Valencia, Spain; smramirez@javerianacali.edu.co; 2 Department of Natural Sciences and Mathematics, Pontificia Universidad Javeriana Cali, 760031 Cali, Colombia; 3 ITACA Institute, Universitat Politècnica de València, Camino de Vera, s/n 46022 Valencia, Spain; aperles@disca.upv.es; 4 Department of Applied Physics (U.D. Agriculture Engineering), Universitat Politècnica de València, Camino de Vera, s/n 46022 Valencia, Spain

**Keywords:** ARIMA, art conservation, Holt–Winters, sensor diagnosis, sparse PLS-DA, TGARCH

## Abstract

The famous Renaissance frescoes in Valencia’s Cathedral (Spain) have been kept under confined temperature and relative humidity (RH) conditions for about 300 years, until the removal of the baroque vault covering them in 2006. In the interest of longer-term preservation and in order to maintain these frescoes in good condition, a unique monitoring system was implemented to record both air temperature and RH. Sensors were installed at different points at the vault of the apse during the restoration process. The present study proposes a statistical methodology for analyzing a subset of RH data recorded by the sensors in 2008 and 2010. This methodology is based on fitting different functions and models to the time series, in order to classify the different sensors.The methodology proposed, computes classification variables and applies a discriminant technique to them. The classification variables correspond to estimates of model parameters of and features such as mean and maximum, among others. These features are computed using values of functions such as spectral density, sample autocorrelation (sample ACF), sample partial autocorrelation (sample PACF), and moving range (MR). The classification variables computed were structured as a matrix. Next, sparse partial least squares discriminant analysis (sPLS-DA) was applied in order to discriminate sensors according to their position in the vault. It was found that the classification of sensors derived from Seasonal ARIMA-TGARCH showed the best performance (i.e., lowest classification error rate). Based on these results, the methodology applied here could be useful for characterizing the differences in RH, measured at different positions in a historical building.

## 1. Introduction

**Figure 1:**
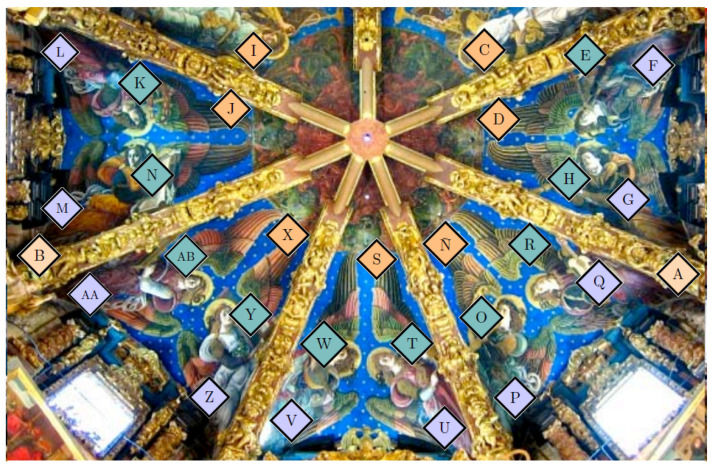
Approximate location of the probes and sensors at the apse vault of the cathedral of Valencia. Details of the installation of the probes and a scheme depicting the installation of probes in the frescoes can be seen in [1] and [2]. The image shows the position of the 29 probes for monitoring the relative humidity (RH) of the indoor atmosphere, displayed in different colors according to their position. Seven probes were located on the ribs (orange), two at the cornice (light orange), ten on the walls below the severies (purple), and ten probes on the frescoes (green).

Over the past 300 years, the famed Renaissance frescoes in Valencia’s cathedral were kept under confined conditions because they were covered by a baroque vault. However, this vault was removed in 2006 [1]. In the interest of longer-term preservation and in order to maintain these frescoes in good condition, a monitoring system was implemented to record both air temperature and relative humidity (RH). Sensors were located at different points in the apse vault. The approximate location of each sensor can be seen in Figure 1. The positions are: cornice C, ribs R, walls W, and frescoes F. Some sensors were inserted on the painting’s surface itself. It is a unique system because sensors are rarely placed inside the frame or on the canvas of paintings. Details about the installation of probes in the frescoes can be seen in figures from [1,2]. A perspective of the upper part of the apse and terrace above the frescoes can be found in [1].

The system was intended to detect water entering from the roof at specific points, or excessive general humidity in the vault itself. Any indication of high levels of thermo-hygrometric conditions would instigate corrective measures [2]. The data analysis carried out by Zarzo et al. [1] showed the advantages of using humidity sensors for the monitoring of frescoes, so as to maximize their protection and prevent deterioration. The microclimatic requirements for churches and cathedrals are similar to those of museums, which also contain valuable works of art [2]. As in the case of museums, the indoor thermo-hygrometric conditions should be maintained at optimal levels in order to conserve the artefacts. The risks constituted by ventilation systems, air-conditioning, central heating, and the presence of visitors should be assessed in order to prevent or slow down the process of deterioration. Ideally, the temperature of walls and their surfaces should be the same as the air in the immediate proximity because, otherwise, an airflow is generated along the wall surface that increments the aerodynamic deposition of airborne particles and wall soiling. Cultural heritage sites are subjected to climatic changes that put them at risk, which has been widely discussed in [3,4].

The internal environment should be appropriate [5] because changes in air temperature and RH can affect the conservation of fresco paintings [6,7,8]. Different studies [9,10,11,12] have monitored thermo-hygrometric parameters inside museums in order to assess the potential risks related to temperature and humidity. Other authors, such as Camuffo et al. [8], have studied the interactions between the indoor atmosphere and walls supporting frescoes or mural paintings. Similar works have been carried out in churches [13,14,15,16,17]. Frasca et al. [13] performed a microclimatic monitoring of the historic church of Mogiła Abbey to analyze the impact of the environmental parameters on the works of art. Among their results, they found that vulnerable objects were at a high risk of mechanical damage approximately 15% of the time. The main cause of the vulnerability was the RH variability.

The problems of deterioration due to high humidity identified in the Renaissance frescoes at the cathedral of Valencia were studied by Zarzo et al. [1]. The researchers suggested that these problems could be caused by the infiltration of rainwater through the roof above the apse and, that maintenance or regular monitoring should therefore be conducted for the long-term preservation of the valuable frescoes. Bernardi et al. [18] studied the importance of waterproofing in the roof above frescoes in St. Stephan’s church in Nessebar, Bulgaria.

In the same way, the European Standards [19,20,21,22,23,24] summarized in the standard [25] as well as Corgnati and Filippi [26] adopted the approach of the Italian Standard UNI 10829 (1999) for the monitoring, elaboration, and analysis of microclimatic data for the preservation of artefacts.

A big economic effort is being carried out by governments within the European Union to preserve artworks in museums. Several works have monitored the microclimate within museums to analyze its relationship with the degradation of materials from which works of art are made, for example, with the goal of preserving artwork and artefacts [27].

Concerning data analysis, García-Diego and Zarzo [2] used monthly principal components analysis (PCA) in their research for February, September, October, and November of 2007. Furthermore, Zarzo et al. [1] also fitted a PCA per year for the years 2007, 2008, and 2010. The resulting loading plots highlight the most relevant similarities and dissimilarities among sensors. Regarding RH recorded in 2007, researchers observed that the daily evolution versus time of the RH mean per hour ($\bar{RH}$) was rather parallel for all sensors. It was observed that sensors H, N, and R (inserted on the frescoes) recorded higher values of $\bar{RH}$ than those installed on the walls. Interestingly, sensors H and R were located in the zone where there was a moisture problem after their installation. In 2008 and 2010, the correlation between $\bar{RH}$ and the first principal component PC1 was very high (greater than 0.994) [1]. After computing the average of moving ranges with order 2 of RH (per hour HMV, day DMV and month MMV), it was shown that PC2 for 2008 could be predicted as: $PC2=232.88-2.69\bar{RH}-32.39DMV$. Regarding 2010, the estimated regression model was: $PC2=297.03-3.87\bar{RH}-32.12DMV$. Based on the results, researchers concluded that PC1 could be interpreted as the yearly RH average, while PC2 provided basic information about daily mean variations. Furthermore, researchers detected an abnormal performance in one sensor that might correspond to a failure of the monitoring system [28] or a change in the microclimatic conditions surrounding that particular sensor. They also concluded that the use of humidity sensors and the interpretation of the first two principal components can be very useful when discussing the microclimatic air conditions surrounding fresco paintings. Hence, PCA is a powerful statistical method for characterizing the different performance among sensors of the same type, located at different positions [1]. The advantage of PCA for sensor diagnosis has also been reported by Dunia et al. [28] and Zhu et al. [29].

The present study re-analyzes time series of RH from sensors located at the apse vault of Valencia cathedral. The data sets used here correspond to subsets of the database used in the study conducted by Zarzo et al. [1]. The present work focuses on RH measurements recorded from 23 sensors in 2008 and from 20 sensors in 2010. These time series of RH do not contain missing values.

This research aims to bring forward a methodology for discriminating sensors according to their position. For this purpose, the approach applied in this study consists of three stages: (1) The different time series were divided according to climatic condition and changes of the slope and level of the time series; (2) Three methods (M1, M2, and M3) were applied to obtain the classification variables per part of the time series identified in stage 1; (3) Sparse partial least squares discriminant analysis sPLS-DA was applied three times (one per method) as a discriminant technique in order to classify sensors, by using the set of classification variables as predictors.

The methodology proposed in this research is new in the context of time series clustering, as well as in sensor classification, when applied with the aim of conserving works of art. This methodology is unique because it uses a Seasonal ARIMA-TGARCH model to extract information from the time series, for discrimination purposes. It is also singular because it employs sPLS-DA in order to classify the time series.

According to the results of this study, sPLS-DA together with ARIMA-TGARCH-Student has a high capability of classifying time series with very similar characteristics, which often occurs in museums or similar buildings. The proposed methodology is well-suited to monitoring the sensors in this type of building.

This approach can be very useful in defining how microclimatic measurements should be carried out for monitoring conditions in heritage buildings or similar sites. Furthermore, the methodology could be useful for reducing the number of sensors required to monitor the microclimate. In summary, this approach could help to better manage the preventive conservation of cultural heritage sites.

## 2. Materials and Methods

### 2.1. Materials: Description of the Data Sets

Regarding the frescoes in Valencia’s cathedral, 29 probes were implemented to monitor the indoor air conditions. Each probe contains an integrated circuit model DS2438 (Maxim Integrated Products, Inc., San Jose, CA, USA) that incorporates an analogue-to-digital voltage converter. This converter measures the output voltage of a humidity sensor (HIH-4000, Honeywell International, Inc., Wabash, IN, USA) and a temperature sensor. The recorded values of RH have an accuracy of ±3.5%. Details of the probes, RH sensors, functions of calibration, and their installation in the apse vault are described elsewhere [1,2]. Seven probes were placed on the ribs (R), two at the cornice (C), ten on the walls below the severies (W), and ten on the frescoes (F) (see Figure 1).

The data sets used here do not contain missing values and correspond to subsets of the database used by Zarzo et al. [1]. The electronics platform Arduino was used https://www.arduino.cc/en/Guide/Introduction. Such data sets correspond to the mean RH per hour or day (${\mathrm{RH}}_{h}$ or ${\mathrm{RH}}_{d}$), where $RH_{h_{t}}$ is the average of measurements per hour, while $RH_{d_{t}}$ corresponds to the average of measurements per day.

The RH datasets correspond to those sensors located in the cornice C, ribs R, walls W, and frescoes F. As the statistical analysis was performed separately for each season, sensors M in W and Ñ at the R were discarded because it was necessary to deal with time series comprising a time period of at least 300 observations without missing values. Thus, time series are available in 2008 for 23 sensors: two on the cornice (A and B), five at the ribs (C, D, I, J, and X), nine on the frescoes (E, H, K, O, R, T, W, Y, and AB), and seven on the walls (G, L, P, U, V, Z, and AA). In 2010, information from sensors H, Y, AB, G, and Z was not available, but there were two additional sensors (S and Q) located at the C and W, respectively. Hence, 20 sensors could be used for 2010: 8 at position RC (R or C), 6 on the walls (W), and 6 on the frescoes (F) (see Figure 1).

For both years, the follow-up time spanned the seasons of winter Wr, spring Sp, and summer Sm. The data set ${\mathrm{RH}}_{h}$ for 2008 consists of 3851 observations: 1430 for Wr, 2099 for Sp, and 322 for Sm. Regarding 2010, 3414 observations are available: 636 for Wr, 2178 for Sp, and 600 for Sm.

Autumn was not considered because the number of observations was not high enough according to the established conditions of the study (i.e., at least 300 observations). Data sets correspond to the periods between 15 January and 4 July 2008, and from 22 February to 18 July 2010. Sensors from the selected times—both in 2008 and 2010—did not experience electronic malfunction. In 2008, there was no evidence of conservation problems in the frescoes where these sensors were located. By contrast, in 2010 there was evidence of salt efflorescence found in the same zones, before the first restoration works in the apse vault [1].

The periods corresponding to each season were defined as follows: spring was considered as being between 19 March and 20 June, summer between 20 June and 22 September, and winter between 22 September and 22 January.

### 2.2. Statistical Methods

Three different methods (M) were applied to the RH data in order to extract estimates of parameters and features used subsequently as classification variables. These methods consist of fitting the time series to different statistical models or functions. M1 included functions such as spectral density, sample autocorrelation function (ACF), sample partial ACF (PACF), and moving range (MR) [30,31,32,33]; M2 was the Additive Seasonal Holt–Winters method (Additive SH-W) [34,35]; and M3 was a Seasonal ARIMA with threshold generalized autoregressive conditional heteroskedastic (TGARCH) model considering the Student distribution for residuals (Seasonal ARIMA-TGARCH-Student) [36,37,38,39].

The three methods were carried out separately for various seasons of the year (Wr, Sp, and Sm) for both 2008 and 2010. Once the classification variables were computed from the three methods, sparse partial least square discriminant analysis (sPLS-DA) [40] was applied to classification variables three times—one time per method—using all classification variables per season. sPLS-DA was used to discriminate between sensors according to their three possible positions in the vault: RC (R or C), W, and F. The classes R and C were joined as a new class called RC in order to ensure a similar number of sensors per group.

The statistical methodology applied consisted of different steps: Firstly, the identification of structural breaks in the time series (Section 2.2.1), which leads to the establishment of periods where the analyses were carried out. Secondly, calculation of classification variables using M1 (Section 2.2.2). Thirdly, the calculation of classification variables using M2 (Additive SH-W; Section 2.2.3). Fourthly, the calculation of classification variables using M3 (Seasonal ARIMA-TGARCH-Student; Section 2.2.4). Finally, sensor classification was done by means of sPLS-DA (Section 2.2.5).

R software [41] version 4.03 was used to carry out the analyses. The main R packages used were aTSA [42], forecast [43,44], mixOmics [45,46], rugarch [37,47], strucchange [48], tseries [49], and QuantTools [33].

#### 2.2.1. Identification of Structural Breaks in the Time Series

Many time series models (e.g., ARMA [36], ARCH, and GARCH [37]) assume the lack of sudden changes due to external factors that might appear occasionally. However, when analyzing time series of real situations, it can be found that external factors produce dramatic shifts such as a change in the slope of a linear trend, which cannot be properly modeled. Such occasional events are known as structural breaks [38]. In order to detect such events, different tests can be applied from the generalized fluctuation test framework (e.g., CUSUM and MOSUM), which are based on empirical fluctuation processes [50]. Others like the Chow [51] test are based on checking sequences of F statistics [52,53,54], while the supF test [55] consists of applying the former at all possible structural breaks. The null hypothesis is “no structural change”, versus the alternative: “the vector of coefficients varies over time” [55].

**Figure 2:**
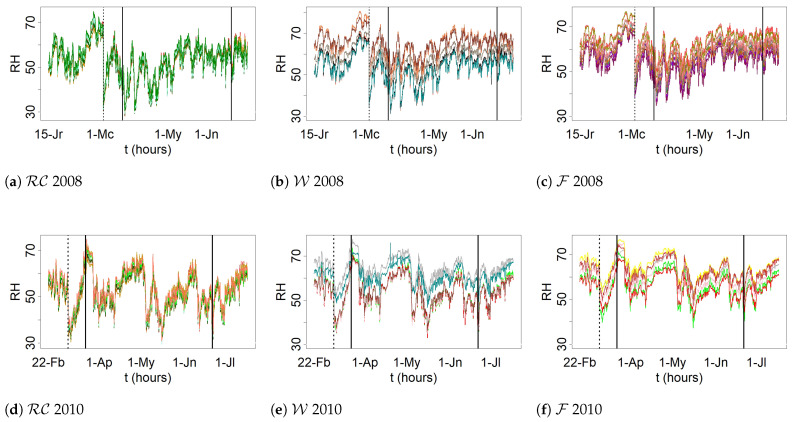
Evolution of ${\mathrm{RH}}_{h}$. Trajectories of sensors located at equivalent positions in the apse vault are depicted in the same chart (data recorded between 15 January and 4 July 2008): cornice and ribs (RC) (**a**), walls (W) (**b**), and frescoes (F) (**c**). Likewise for data collected between 22 February and 18 July 2010: RC (**d**), W (**e**), and F (**f**). Separation by seasons (Wr, Sp and Sm) is indicated by means of vertical solid lines. Wr is divided into two periods (dashed line) because a structural break was identified according to the supF and CUSUM tests.

**Figure 3:**
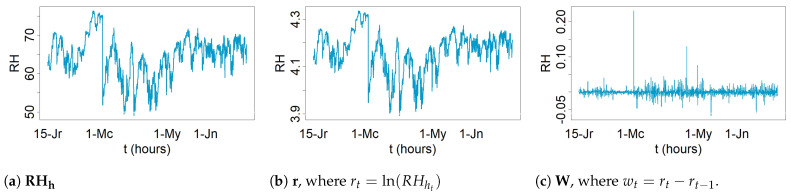
(**a**) Observed time series of RH from sensor Y (2008), (**b**) logarithmic transformation of the time series of (**a**), (**c**) one regular differentiation of the time series of (**b**).

By visually inspecting the evolution versus time of ${\mathrm{RH}}_{h}$ for both 2008 and 2010 (see Figure 2), potential structural breaks were identified in at least two points. Their significance was assessed by means of the CUSUM and supF tests. Both tests were carried out with the logarithmic transformation [56], that is, $r_{t}=\ln\left(RH_{h_{t}}\right)$, which has been used in other works to stabilize the data variance [38,56,57]. It was observed that most of the daily time series undergo seasonal trends, which makes it necessary to apply regular differentiation (i.e., $w_{t}=r_{t}-r_{t-1}$) in order to remove any trend [56], which is a common pretreatment in time series analysis. In this paper, r refers to transformed values using the logarithmic function, while W refers to data that was subjected to a logarithmic transformation and one regular differentiation. Figure 3 displays the plot of the time series of RH from sensor Y (2008). Additionally, this figure shows the plots of the time series of the logarithmic transformation of RH as well as one regular differentiation of the previous time series.

The supF and CUSUM tests were applied to six groups: Wr 2008 (group 1, n=1429), Sp 2008 (group 2, n=2098), Sm 2008 (group 3, n=321), Wr 2010 (group 4, n=635), Sp 2010 (group 5, n=2177), and Sm 2010 (group 6, n=559).

According to the supF test, a structural break was identified in Wr 2008, after the 1058th observation (27 March at 7:00 a.m., *p*-value = 0.01). Another break was found in 2010 at the 338th observation (8 March at 1:00 p.m., *p*-value = 0.04). The CUSUM chart identified a significant shift at the same instant of time (1058th value in 2008 and 338th observation in 2010). The main reason for structural breaks could have been the strong changes of RH that occur in Valencia.

Ignoring structural breaks can lead to negative implications such as inconsistency of the parameter estimates and forecast failures [58]. Accordingly, for each structural break, it was decided to fit one model before this event, and another one after the structural break. On the other hand, in congruence with the physical characteristics of the data, it might be convenient to split the statistical analysis per season and year. According to both considerations, the analysis was carried out separately in four periods, denoted as WrA, WrB, Sp, and Sm. WrA corresponds to the winter period before the structural break, while WrA refers to the following period (see Figure 2).

#### 2.2.2. Calculation of Classification Variables—Method M1

**Figure 4:**
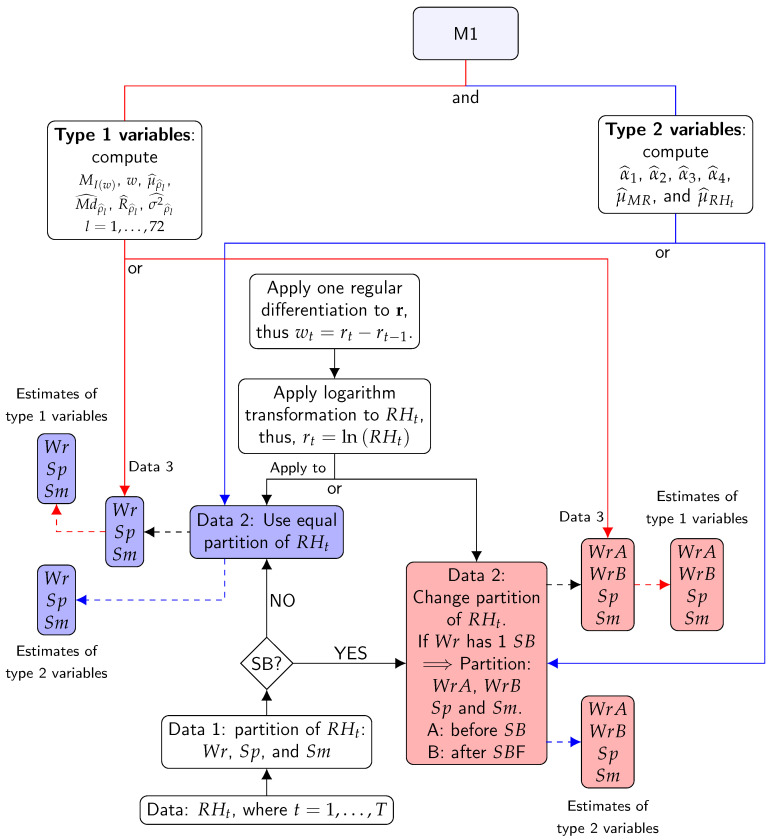
Flow chart for the steps of method 1: Blue lines indicate type 2 variables. Red lines indicate type 1 variables. Solid lines indicate processes. Dashed lines indicate results. The first step consists of dividing the different time series according to the climatic conditions: Wr, Sp, and Sm (Data 1). The second step consists of dividing the time series (Data 1) according to possible structural breaks (SBs) (Data 2). The third step applies a logarithm transformation and one regular differentiation to Data 2. The result is Data 3. The fourth step consists of applying the formulas of type 2 variables to Data 2. This is the first result. The fifth stage is carried out by applying the formula of type 1 variables to Data 3. The outcome produced is result 2. Different boxes contain symbols such as Wr, Sp, and Sm (or WrA, WrB, Sp, and Sm). They indicate that the results were computed for all different parts of the time series.

This method is based on features using estimates of ACF ($\rho_{l}$ at lag *l*), PACF ($\alpha_{l}$ at lag *l*), as well as features using mean (μ) and moving range (MR). Furthermore, features from spectral density were used, which was estimated using the periodogram (I(w)) of signals *w*. These features can help to characterize and reveal interesting properties of the underlying stochastic process without using any specific parametric model. Figure 4 shows a summary of the steps of M1.

RH values were used for estimating PACF, mean, and MR. By contrast, logarithm transformation and regular differencing were applied before estimating ACF and spectral density in order to stabilize the variances and remove the trend (i.e., W was employed). The objective of using both the ACF and spectral density with W was to focus on the seasonal component of the time series. These functions are briefly explained below:

Firstly, the mean of ${\mathrm{RH}}_{h}$ was estimated for each period because this variable appeared as important for discrimination purposes in the preliminary study [1,2].

Secondly, MR with order *n* correspond to range values over *n* past values [33]. This function was applied to ${\mathrm{RH}}_{h}$ and ${\mathrm{RH}}_{d}$. For each period, the mean and variance were computed for all MR values with order 2. These variables were calculated in order to estimate HMV and DMV, which were used in the preliminary investigations of this project [1,2]. However, MMV (i.e., MR of order 2 for ${\mathrm{RH}}_{m}$, estimated with the average RH per month) could not be calculated in this research because the number of observations per month was too low. For ${\mathrm{RH}}_{h}$, the mean of MR (${\hat{\mu }}_{MR}$) corresponds to HMV and for ${\mathrm{RH}}_{d}$, the mean of MR is represented by DMV.

Thirdly, spectral density was estimated by means of the periodogram, which was calculated on the log scale using a spectrum function [30]. The periodogram displays information about the strengths of the various frequencies for explaining the seasonal components of a time series. The maximum peaks of spectral density and their corresponding frequencies were identified [30]. These functions were applied to ${\mathrm{RH}}_{h}$.

Finally, an estimation of ACF at lag *l* is the correlation (quantified by means of Pearson’s correlation coefficient) between the values of a given time series, with the lagged values of the same time series at *l* time steps (*l* refers to lags) [30]. W values were used for this calculation. The values of sample ACF for the lags from 1 to 72 were used as classification variables because they showed greater variations, while for further lags they displayed lower values close to zero, comprised between the limits of a 95% confidence interval in the ACF correlogram.

Regarding sample PACF, according to Cowpertwait and Metcalfe [59], “the partial autocorrelation at lag *l* is the correlation that results after removing the effect of any correlations due to the terms at shorter lags”. Sample ACF and sample PACF plots are commonly used in time series analysis and forecasting (e.g., autoregressive moving average (ARMA) models and their particular cases such as autoregressive (AR) and moving average (MA) models [59]). These plots, also called correlograms, illustrate the strength of a relationship between the values observed at a certain instant of time with those recorded in previous moments (with lag *l*) in the same time series. If sample ACF values decline exponentially and there are spikes in the first or more lags of sample PACF values, the time series can be modeled as an AR process. If sample PACF values decline exponentially and there are spikes in the first or more lags of the sample ACF values, the time series can be modeled as an MA process. If both sample PACF and sample ACF values decline exponentially, the time series can be modeled as a mixed ARMA process [59]. Sample PACF was computed for ${\mathrm{RH}}_{h}$ values. The sample PACFs for the first four lags were calculated for each period and were regarded as classification variables because they are usually the most important ones for capturing the relevant information in time series.

The features computed using the values of RH were called type 1 variables and features calculated using values of W were referred to as type 2 variables. The list of type 1 variables resulting from M1 are the estimates of the following parameters:

- Mean of ${\mathrm{RH}}_{h}$ (${\hat{\mu }}_{RH}$).
- Mean of MR (${\hat{\mu }}_{MR}$) of order 2 for ${\mathrm{RH}}_{d}$ and ${\mathrm{RH}}_{h}$.
- Variance of MR (${\hat{\sigma^{2}}}_{MR}$) of order 2 for ${\mathrm{RH}}_{d}$ and ${\mathrm{RH}}_{h}$.
- PACF for the first four lags (${\hat{\alpha }}_{1}$, ${\hat{\alpha }}_{2}$, ${\hat{\alpha }}_{3}$, and ${\hat{\alpha }}_{4}$).

The list of type 2 variables resulting from M1 are the estimates of the following parameters:

- Maximum of spectral density ($M_{I(w)}$) and frequency corresponding to the maximum (*w*).
- Mean (${\hat{\mu }}_{{\hat{\rho }}_{l}}$), Median (${\hat{Md}}_{{\hat{\rho }}_{l}}$), range (${\hat{R}}_{{\hat{\rho }}_{l}}$), and variance of the sample ACF (${\hat{\sigma^{2}}}_{{\hat{\rho }}_{l}}$) for the first 72 lags.

#### 2.2.3. Calculation of Classification Variables—Method M2: Additive SH-W

Winters [35] extended Holt’s method [34] for capturing the seasonality of a time series. Hence, it was called Holt–Winters (H-W), which is a particular method of exponential smoothing aimed at forecasting [60].

The Seasonal H-W approach (SH-W) is based on three smoothing equations: for the level, trend, and seasonality. The parameter *S* denotes the number of values per season, while three additional parameters capture the information at time *t*: $a_{t}$ denotes the time series level, $b_{t}$ is the slope, and $s_{t}$ is the seasonal component [60]. There are two different SH-W methods, depending on whether seasonality is modeled additively or multiplicatively [60]. The Seasonal H-W approach (SH-W) is based on three smoothing equations: for the level, trend, and for seasonality. The parameter *S* denotes the number of values per season, and three additional parameters capture the information at time *t*: $a_{t}$ denotes the time series level, $b_{t}$ is the slope, and $s_{t}$ is the seasonal component [60].

**Figure 5:**
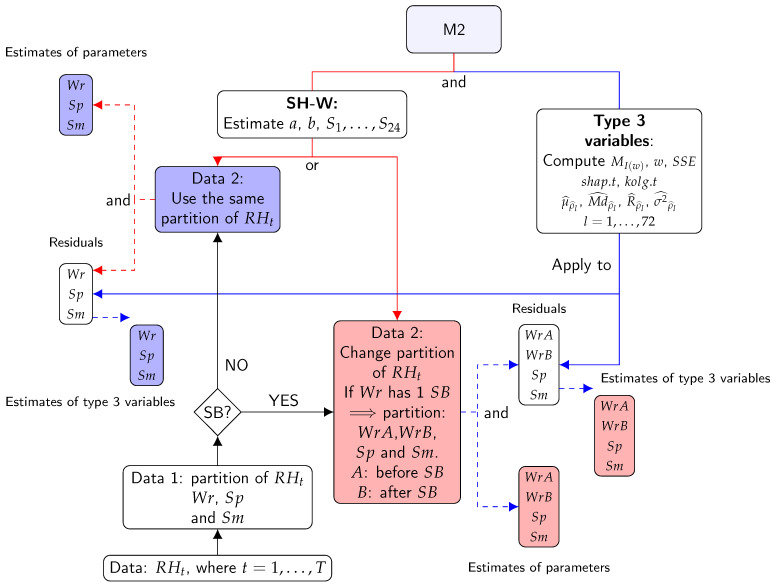
Flow chart for the steps of method 2: Red lines indicate estimated parameters. Blue lines indicate type 3 variables. Solid lines indicate processes. Dashed lines indicate results. The first step divides the different time series according to the climatic conditions ( Wr, Sp, and Sm) (Data 1). The second step organises the time series according to possible structural breaks (SBs) (Data 2). The third step applies the method to Data 2 in order to obtain the estimates of the method’s parameters (first result) and then the residuals from the method. The fourth step consists of applying the formulas for type 3 variables to the residuals (second result). Different boxes display symbols Wr, Sp, and Sm (or WrA, WrB, Sp, and Sm). This indicates that the results correspond to all different parts of the time series.

In this research, the Additive SH-W method was fitted to both time series of RH and to their logarithmic transformations, but it turned out that the best outcomes were obtained with the transformed data. The period, the number of observations per season, was considered as S=24 (i.e., 24 hourly values per day). Although this method does not require a residual analysis, one was carried out in an attempt to extract further information. Autocorrelation within the time series appeared in at least 10 out of the 22 lags for over 80% of the Ljung–Box Q (LBQ) tests [61] applied. Furthermore, the Kolmogorov–Smirnov normality (KS normality) [62] and Shapiro–Wilk (SW) tests [57,63,64] rejected the hypothesis of normality for at least 80% of the cases applied. The KS normality test compares the empirical distribution function with the cumulative distribution function. The test statistic is the maximum difference between the observed and theoretical values (normality). The statistic of the KS normality test was used as a classification variable in order to gather information about the distribution pattern of residuals and quantify departure from a normal distribution. The SW test detects deviations from normality due to either skewness or kurtosis, or both. The statistic of the SW test was also employed as a classification variable in order to identify lack of normality in the residuals according to skewness and kurtosis. Furthermore, given that data sets in this study are seasonal with a period of 24 h, where 72 is the maximum of lags, this maximum value was also considered for estimation of the mean, median, range, and variance of the sample ACF for the residuals. Figure 5 shows a summary of the steps of M2.

The features computed from residuals of the SH-W method were called type 3 variables. The other classification variables corresponded to the estimates of the method’s parameters. The list of classification variables resulting from M2 are the following:

- Estimates of the parameters of the SH-W method: trend ($\hat{b}$), level ($\hat{a}$), and seasonal components (${\hat{S}}_{1},\ldots ,{\hat{S}}_{24}$).
- type 3 variables: sum of squared estimate of errors (SSE), maximum of spectral density (${\hat{M}}_{I(w)}$), frequency corresponding to maximum of spectral density ($\hat{w}$), and the mean (${\hat{\mu }}_{{\hat{\rho }}_{k}}$), median (${\hat{Md}}_{{\hat{\rho }}_{k}}$), range (${\hat{R}}_{{\hat{\rho }}_{k}}$), and variance (${\hat{\sigma^{2}}}_{{\hat{\rho }}_{k}}$) of sample ACF for 72 lags. The statistic of the SW test (shap.t), and the statistic of the KS normality test (kolg.t) are also included in this list.

#### 2.2.4. Calculation of Classification Variables—Method M3: Seasonal ARIMA-TGARCH-Student

ARMA models were popularized by Anderson [65], who developed a coherent three-step iterative cycle for time series estimation, verification, and forecasting. This method is also known as the Box–Jenkins approach. The ARMA model assumes that the time series is stationary; if this is not the case, differencing the time series one or more times is required, resulting in an ARIMA model. In the ARIMA(p,d,q) approach, *p* is the number of AR terms, *d* is the number of regular differences taken, and *q* is the number of the MA. Furthermore, $\phi_{i}$ (i=1,⋯,p) are the parameters of the AR part of the model, $\theta_{j}$ (j=1,⋯,q) are the parameters of the MA part, and the $\varepsilon_{t}$ are error terms—generally assumed to be a white noise sequence [38].

Although ARIMA is flexible and powerful in forecasting, it is not able to properly handle continuously changing conditional variance or the non-linear characteristics of the variance that can be present in some time series [66]. This is often referred to as variance clustering or volatility [67,68]. If it is assumed that a given time series follows an ARIMA process, the conditional variance of residuals is supposed to be constant versus time. When this condition is not fulfilled, it is known as a conditional variance process [56,67,68]. In such a case, data can also be affected by non-linear characteristics of the variance. These patterns can be studied using the GARCH family of models. Two of the most important ones for capturing such changing conditional variance are the ARCH and generalized ARCH (GARCH) models developed by Engle [69] and later extended by Bollerslev [70]. Engle and Bollerslev [71] were pioneers in the area of volatility modeling by introducing ARCH and, subsequently, GARCH models, which provide motion dynamics for the dependency in the conditional time variation of the distributional parameters of the mean and variance.

In recent years, different studies have applied hybrid forecasting models in various fields, and have shown a good performance for rainfall data [72], for the price of gold [73], for forecasting daily load patterns of energy [74], and for stock market prices [75].

According to Ghalanos [37], the family of GARCH models is broad, including the standard, integrated, and exponential models, as well as the GJR-GARCH, the asymmetric power ARCH, and the threshold GARCH (TGARCH) of Zakoian [76]. They capture the asymmetry of occasional impacts as well as abnormal distributions to account for the skewness and excess kurtosis. For GARCH models, error terms can sometimes be assumed from the Student distribution [77]. Bollerslev [78] described the GARCH-Student model as an alternative to the normal distribution for fitting the standardized time series. In particular, in the TGARCH-Student(s,r) model, *s* is the number of GARCH parameters $\beta_{i}$ (i=1,⋯,s), *r* is the number of ARCH and rotation parameters $\alpha_{j}$ and $\eta_{1j}$, respectively (j=1,⋯,r), while ω is the variance intercept parameter. Error terms $\epsilon_{t}$ are assumed to be a white noise sequence following a Student distribution with degrees of freedom *v* [37].

Thus, instead of considering the standard ARIMA approach, whose focus is the conditional mean, it seems convenient to use here a hybrid approach based on ARIMA and GARCH models which can simultaneously deal with both the conditional mean and variance [38].

Given that data sets in this study are seasonal, it is necessary to use Seasonal ARIMA models, which are capable of modeling a wide range of seasonal data. A Seasonal ARIMA$\left(p,d,q\right){\left(P,D,Q\right)}_{S}$ model is characterized by additional seasonal terms: *P* is the number of seasonal AR (SAR) terms, *D* is the number of differences taken, *Q* is the number of seasonal MA (SMA) terms, and *S* is the number of observations per period (S=24 in this study). Furthermore, $\Phi_{i}$ (i=1,…,P) are the parameters of the SAR part of the model, $\Theta_{j}$ (j=1,…,Q) are the parameters of the SMA part, and the $\varepsilon_{t}$ are error terms, which are assumed to be a white noise sequence [38]. In particular, in the Seasonal ARIMA$\left(p,d,q\right){\left(P,D,Q\right)}_{S}$ -TGARCH(s,r) -Student model, the errors $\varepsilon_{t}$ from Seasonal ARIMA$\left(p,d,q\right){\left(P,D,Q\right)}_{S}$ follow a TGARCH(s,r) -Student process of orders *s* and *r*, so that their error terms $\epsilon_{t}$ are assumed to be a white noise sequence following a Student distribution, with degrees of freedom *v*.

Two steps were considered for the application of a hybrid approach based on Seasonal ARIMA and GARCH models, as briefly explained below.

Firstly, the most successful Seasonal ARIMA (or ARIMA) model was selected and the residuals were computed. Next, the most successful GARCH model was applied to fit these residuals. The following steps were carried out:

- The condition of stationarity was checked, that is, whether the statistical characteristics of the time series were preserved across the time period. The null hypothesis was that mean and variance do not depend on time *t* and the covariance between observations $RH_{t}$ and $RH_{t+l}$ does not depend on *t* [38]. To examine this null hypothesis, the augmented Dickey–Fuller (ADF) [79] and LBQ tests were applied for 48 lags. Furthermore, the sample ACF and sample PACF plots were also used.
- Transformation and differencing: the logarithmic transformation and regular differentiation were applied to ${\mathrm{RH}}_{h}$ data before fitting ARMA in order to transform nonstationary data into stationary data [59]. The criterion for determining the values of *d* ( differencing) is explained in the next step. The logarithmic transformation was preferred over other transformations because the variability of a time series becomes more homogeneous using logarithmic transformation, which leads to better forecasts [80].
- Identification of the most appropriate values for (p,d,q) and (P,D,Q). Sample ACF and sample PACF plots were used to identify the appropriate values of (p,q). Furthermore, the *corrected Akaike information criterion* (AICc) [60] was useful for evaluating how well a model fits the data and determining the values of both (P,D,Q) and (p,d,q), taking into account the restriction that *d* and *D* should be 0 or 1. The most successful model for each time series was chosen according to the lowest AICc value. The AICc values were compared for models with the same orders of differencing, that is, equal values of *d* and *D*.

Secondly, the maximum likelihood estimation (MLE) method was used for estimating the parameters of the Seasonal ARIMA (or ARIMA) [38]. The models chosen were statistically examined in order to ensure that the resulting residuals do not contain useful information for forecasting. For this purpose, different tests were applied to determine whether all conditions and model assumptions were fulfilled. The analysis of residuals was carried out as follows:

- The condition of white noise was checked. Error terms can be regarded as white noise if their mean is zero and the sequence is not autocorrelated [38]. In order to check this issue, the ADF and LBQ tests were applied to the residuals and their squared values for 48 lags. Furthermore, the sample ACF plots were also used.
- To study the absence of Arch effects: for this purpose, the Lagrange multiplier test [69] and sample ACF plots [67] were applied to the residuals and their squared values [81].
- To check the distribution of residuals: by means of the Q–Q normal scores plots as well as the SW and KS normality tests.

The analysis of residuals revealed that error terms follow a GARCH process in all the different ARIMA models that were fitted. Therefore, it was necessary to fit a GARCH model to these residuals. The estimated model parameters were checked to determine if they were statistically significant, and their residuals were evaluated as described above. Finally, a hybrid model was fitted for each sensor and each period using the combined Seasonal ARIMA (or ARIMA) and TGARCH approach. After repeating the steps iteratively in order to select the most successful model, the normality tests applied to their residuals rejected the hypothesis of normality in all cases. Furthermore, all Q–Q normal scores plots showed that residuals were not falling close to the line at both extremes. Thus, a Student distribution was used to fit the residuals of the different TGARCH models.

For each period, a common model was applied to the hourly data of each sensor (one day corresponds to a sequence of 24 values).

- WrA (2008): seasonal ARIMA$\left(1,1,0\right){\left(2,0,0\right)}_{24}-$ TGARCH(1,1)-Student.
- WrA (2010): ARIMA(1,1,2)− TGARCH(1,1)-Student.
- WrB (2008 and 2010): seasonal ARIMA$\left(1,1,1\right){\left(2,0,0\right)}_{24}-$ TGARCH(1,1)-Student.
- Sp (2008 and 2010): seasonal ARIMA$\left(1,1,2\right){\left(0,0,2\right)}_{24}-$ TGARCH(1,1)-Student.
- Sm (2008 and 2010): seasonal ARIMA$\left(1,1,1\right){\left(1,0,0\right)}_{24}-$ TGARCH(1,1)-Student.

A seasonal model was not selected for WrA (2010) because the analysis of residuals of the selected model showed similar results to the best seasonal model, and the selected model was simpler.

**Figure 6:**
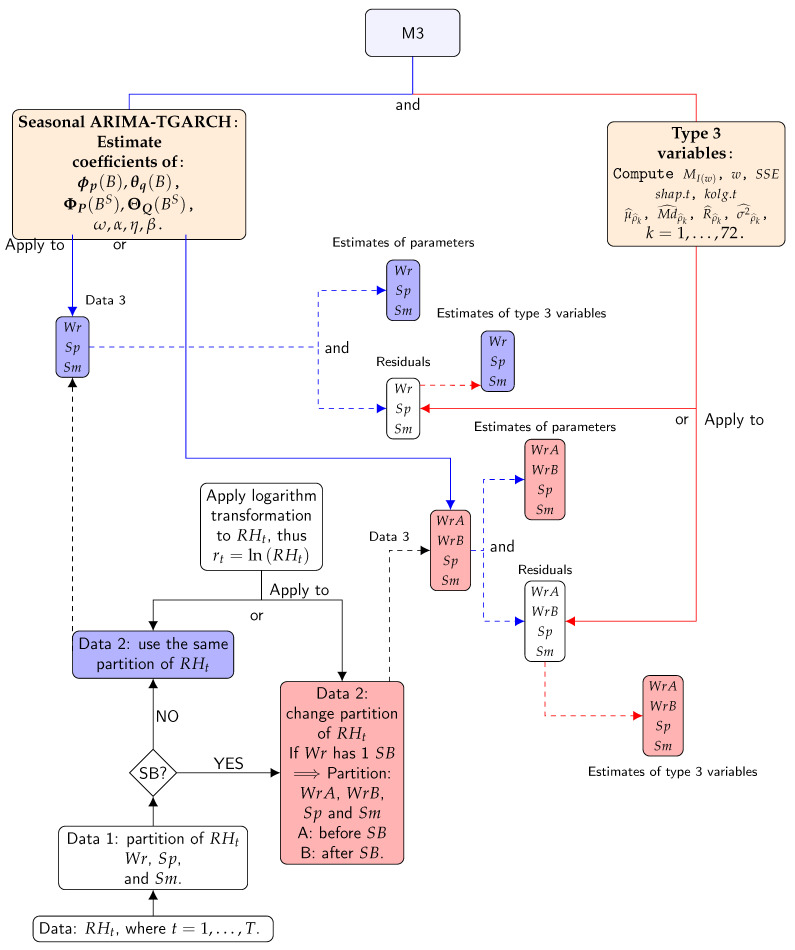
Flow chart for the steps of method 3: Blue lines indicate estimated parameters. Red lines indicate type 3 variables. Solid lines indicate processes. Dashed lines indicate results. The first step divides the different time series according to the climatic conditions (Wr, Sp, and Sm) (Data 1). The second step organises the time series according to possible structural breaks (SBs) (Data 2). The third step applies logarithmic transformation to Data 2, the result is Data. The fourth step applies the model to Data 3 in order to obtain the estimates of model parameters (first result) and then the residuals from the method. The fifth step consists of applying the formulas of type 3 variables to the residuals (second result). Different boxes display symbols Wr, Sp, and Sm (or WrA, WrB, Sp, and Sm). This indicates that the results correspond to all different parts of the time series.

When analyzing the residuals from Seasonal ARIMA-TGARCH-Student models for 2008, it turned out that in the case of WrA, the time series from 12 sensors out of the 23 available did not satisfy all expected conditions. The same happened for Sp: 14 out of the 20 models did not fulfill all requirements. Thus, in an attempt to extract further information not properly captured by these models, some features were calculated from the residuals. Figure 6 shows a summary of the steps of M3.

In all cases, residuals from the ARIMA-GARCH-Student models displayed evidence of stationarity for 48 lags. However, in some cases, there was evidence of autocorrelation as well as the presence of ARCH effect. For the tests applied to residuals, 0.03 was the maximum *p*-value found to reject the null hypothesis. Regarding 2008, the number of time series (from the 23 sensors) that satisfied all tests in the residual analysis, is the following: 12 in WrA, 22 in WrB, 22 in Sp, and 20 in Sm. In 2010, out of the 20 sensors available, the values are: 18 in WrA, 19 in WrB, 14 in Sp, and 15 in Sm.

The features computed from residuals of the models were called type 3 variables. The other classification variables corresponded to the estimates of the parameters of the selected models. The estimates of the parameters are as follows:

- Estimated parameters from ARIMA of: (1) the regular autoregressive operator ($\phi_{p}\left(B\right)$) of order *p* and the regular moving average operator ($\theta_{q}\left(B\right)$) of order *q*: ${\hat{\phi }}_{1}$, ${\hat{\phi }}_{2}$, ${\hat{\theta }}_{1}$, ${\hat{\theta }}_{2}$, etc.; (2) the seasonal autoregressive operator ($\Phi_{P}\left(B^{24}\right)$) of order *P* and the seasonal moving average operator $\Theta_{Q}\left(B^{24}\right)$ of order *Q*: ${\hat{\Phi }}_{1}$, ${\hat{\Phi }}_{2}$, ${\hat{\Theta }}_{1}$, ${\hat{\Theta }}_{2}$, etc.
- Estimated parameters from TGARCH (1,1): $\alpha_{1}$, $\eta_{11}$, $\beta_{1}$, ω, and *v* (for Student distribution).

The estimate of type 3 variables:

- Variance of the residuals ($\hat{\sigma^{2}}$), maximum of spectral density of the residuals (${\hat{M}}_{I(w)}$), frequency corresponding to maximum of spectral density ($\hat{w}$), mean (${\hat{\mu }}_{{\hat{\rho }}_{k}}$), median (${\hat{Md}}_{{\hat{\rho }}_{k}}$), range (${\hat{R}}_{{\hat{\rho }}_{k}}$), and variance (${\hat{\sigma^{2}}}_{{\hat{\rho }}_{k}}$) of sample ACF for 72 lags. The statistic of the SW test (shap.t) and the statistic of the KS normality test (kolg.t) are also included.

#### 2.2.5. Sensor Classification by Means of sPLS-DA

Once all classification variables were computed as described above for the data from 2008, they were structured in three matrices, one per method (denoted as $X_{1}$, $X_{2}$ and $X_{3}$, respectively), with 23 rows (sensors) where the classification variables are in columns. The total number of variables obtained from each method was 53 for $X_{1}$, 141 for $X_{2}$, and 59 for $X_{3}$. Likewise, regarding 2010, classification variables were structured in three analogous matrices with 20 rows and with the same number of variables.

As the number of classification variables is much greater than the number of sensors, this scenario suggests a high degree of multicollinearity, and it might lead to severely ill-conditioned problems. Different approaches can be considered to deal with this problem. One solution is to perform variable selection, or to apply methods based on projection to latent structures like partial least squares discriminant analysis (PLS-DA).

One advantage of this multivariate tool is that it can handle many noisy and collinear classification variables, being computationally very efficient when the number of variables is much greater than the number of sensors. Even though PLS-DA is extremely efficient in a high-dimensional context, the interpretation of results can be complex in the case of a high number of variables. In such a case, sparse PLS-DA (sPLS-DA) has very satisfying predictive performance, and is able to select informative variables easily. Therefore, it was decided to apply sPLS-DA [40] here using the classification data sets mentioned above in order to identify a small subset of components and classification variables aimed at sensor clustering.

The algorithm of sPLS-DA used here was the one proposed by Rohart et al. [45], which corresponds to a modified version developed by Lê Cao et al. [40]. This new version uses the penalty $\ell_{1}$ (lasso) on the loading vector of the regressor matrix by shrinking to zero the coefficient of some variables according to Rohart et al. [45].

With the aim of sensor clustering, sPLS-DA was applied to the previously mentioned classification data sets. Three categories of positions were considered for the sensors: RC, W, and F. This method was applied to the three matrices ($X_{1}$, $X_{2}$, and $X_{3}$) containing the classification variables, with dimension n×p, where *p* is the number of classification variables and *n* is the number of sensors. Furthermore, Y is a vector of length *n* that indicates the class of each sensor, with values coded as 1 (for RC), 2 (F) and 3 (W). This vector has to be converted into a dummy matrix (Z, i.e., with values either 0 or 1) with dimension n×K, where *n* is the number of sensors and K=3 the number of classes or positions of sensors.

Before applying sPLS-DA, all anomalous values of each classification variable were removed and considered as missing data after being previously identified using normal probability plots and box plots for each variable. As a result, in 2008: 1.20% (M1), 1.04% (M2), and 1.06% (M3) were the percentages of missing values of the classification data sets. In 2010, the corresponding percentages were 0.40%, 1.39%, and 0.49%, respectively, for each method. These values are relatively low. Furthermore, all classification variables were normalized (i.e., centered and scaled to unitary variance). The package mixOmics [45] was used to perform sPLS-DA, which is able to handle missing values by using the NIPALS algorithm [45,82].

Three-fold cross-validation (three-fold CV, S1 supplementary information of [45]) was used to evaluate the performance (i.e., low classification error rate) of the PLS-DA. It was used to determine both the optimal number of components and the optimal number of variables. The three-fold CV was performed with stratified subsampling, where all positions (RC, F, and W) are represented in each fold.

In order to select the optimal number of components, three-fold CV was applied for a maximum number of ten components, which was repeated 1000 times for each fold. With the objective of assessing the PLS-DA performance, the classification error rate (CER), the overall classification error rate (denoted as Overall), and the balanced classification error rate (BER) were computed [45]. Each BER value corresponds to the average proportion of wrongly classified sensors in each class, weighted by the number of sensors in each class. BER is less biased towards majority classes during the performance assessment when compared with the Overall criterion [45]. Thus, BER was considered instead of the latter.

The classification of sensors was determined according to different prediction distances (PD): maximum, centroid, and Mahalanobis [45]), which were computed for each sensor. Among the three distances calculated, it was found that the centroid one performed better in most cases for the classification, and hence it was selected. Regarding the centroid distance, the software computed the centroid (*G*) of the learning set of sensors (training data) belonging to the classes (RC, F, and W). Each centroid *G* was based on the *H* latent components associated with X. The distances were calculated from the components of the trained model. The position of the new sensor was assigned according to the minimum distance between the predicted score and the centroids *G* calculated for the three classes considered.

The optimal number of components *H* was achieved by determining the best performance, based on the BER criterion and prediction distances according to the centroid distance. Once the optimal number of components was determined, repeated three-fold CV was carried out to establish the optimal number of variables according to the criteria of centroid and BER. Finally, once the optimal number of components and variables was decided, the final PLS-DA model was computed.

**Figure 7:**
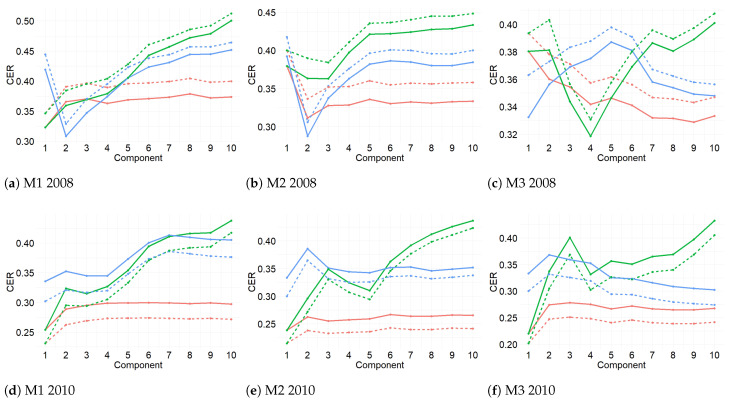
Classification error rate (CER) from PLS-DA for 10 components. The CER was computed for each prediction distance (maximum, centroid, and Mahalanobis) per method (M1, M2, and M3) for 2008 and 2010. Two types of error rate are indicated: balanced BER (dashed lines) or Overall (solid lines). Blue lines refer to maximum distance, red lines to centroid distance, and green lines to Mahalanobis distance. PLS-DA was carried out using repeated three-fold CV 1000 times. For 2010, BER and centroid distance showed the best performance achieved by one component. In 2008, M1 and M2 performed the best for maximum distance and Overall, while Mahalanobis distance performed the best in M3. The second-best distance for the three methods was the centroid distance.

Figure 7 displays the results from the first three-fold CV for the three methods and for both years. For 2010, the values of BER and centroid distance suggested that one component is enough to classify the time series, while for 2008 the results indicate that one or two components are necessary. From this step, the centroid distance and BER were selected in order to determine the number of components.

**Figure 8:**
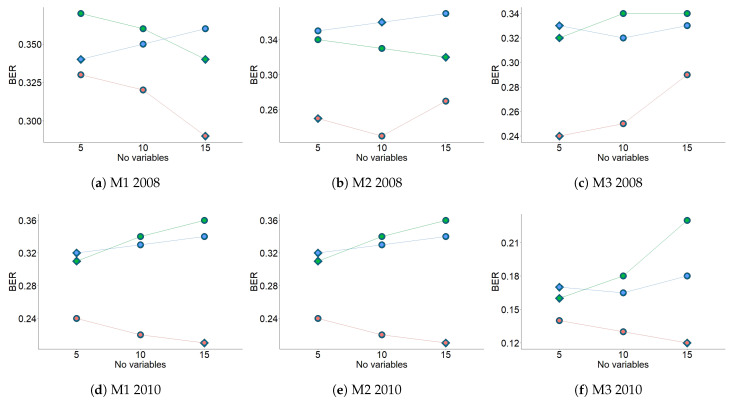
BER according to the number of variables (5, 10 or 15) and different number of components (1: orange dots, 2: blue dots, or 3: green dots) for each method, for 2008 and 2010. Three-fold CV was run 1000 times using centroid distance prediction. Diamonds indicate the optimal number of variables per component according to the lowest value of BER.

**Figure 9:**
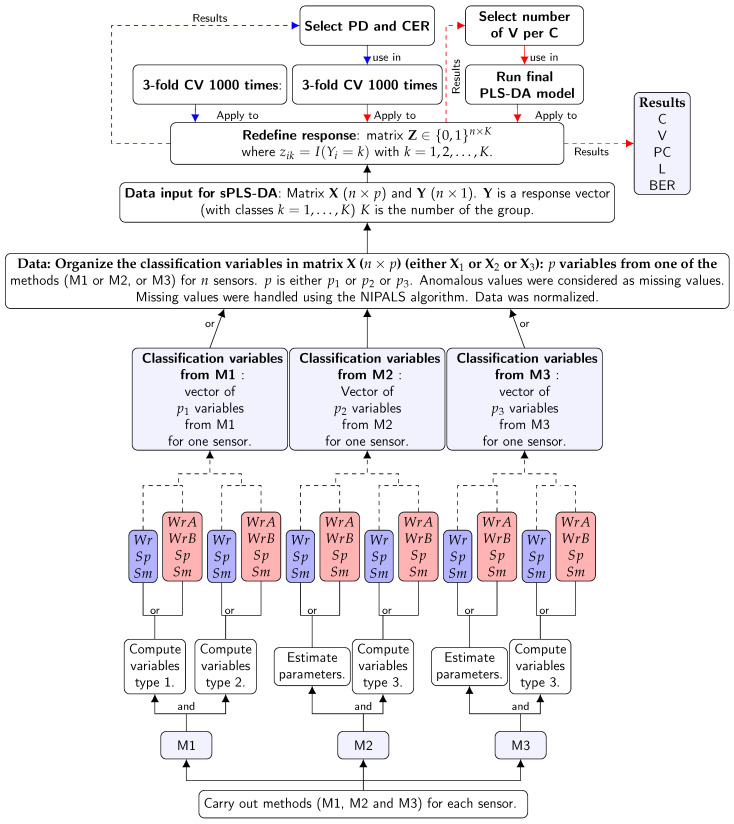
Flow chart for the stages used to apply sparse partial least squares discriminant analysis (sPLS-DA) to the results from the three methods. In the box titled “Data”, the information corresponds to the variables from one of the three methods. If the information is from $M_{i}$ then X=$X_{i}$. The values were computed for all sensors. Thus, a matrix X was obtained. The values were treated before running the sPLS-DA algorithm. In the box titled “Data input for sPLS-DA”, the information corresponds to the response vector converted into a dummy matrix Z. In the following boxes the PLS-DA algorithm runs from left to right. The first three-fold CV was used to evaluate PLS-DA and the prediction distance PD, classification error rate (CER) with the optimal number of components selected. This information was used in the second three-fold CV to check PLS-DA in order to select the optimal number of variables V. The information obtained using both three-fold CVs was used to run the final PLS-DA.

Figure 8 shows the results from the second three-fold CV for the three methods and for both years. For 2008, the results suggested that the number of variables per one component were 15 (M1), 10 (M2), and 5 (M3). For 2010, the results suggest the number of variables per one component was 15 for all methods. The information in Figure 7 and Figure 8 (centroid.dist, BER, and number of variables per component) was used to apply the final PLS-DA. Figure 9 describes the steps used to apply sPLS-DA, using the results from the three methods in the study.

Figure 9 shows the summary of steps of the sPLS-DA.

The main outputs from the analysis are: (1) a set of components (C) associated with $X_{1}$, $X_{2}$, and $X_{3}$ for the matrix Z; (2) a set of loading (L) vectors containing the coefficients assigned to each variable that define each component; (3) a list of selected variables (V) from $X_{i}$ (i=1,2,3) associated with each component; (4) the values of BER for each component; and (5) the predicted class (PC) for each sensor. Coefficients in a given loading vector indicate the importance of each variable.

## 3. Results

Components from sPLS-DA are linear combinations of variables that might correspond to WrA, WrB, Sp, or Sm. By applying sPLS-DA to $X_{1}$, $X_{2}$, and $X_{3}$, only one component appeared to be relevant in all cases. The variables selected (per component) by the sPLS-DA algorithm are indicated in the following paragraph. The final model which used the classification variables from M1 (2008) is based on 15 selected variables. The selected model from M2 (2008) consists of 10 variables, while just 5 variables were considered for M3 (2008). The final model for M1, M2, and M3 (2010) comprises 1 component and 15 selected variables from each model (see Table 1).

The BER values are indicated in Table 1a,b for the three methods, using data from 2008 and 2010, respectively. For both years, the classification variables which turned out to be the most important for the first component were ordered according to the absolute value of their loading weights, from highest to lowest. The notation of the results is ${\hat{M}}_{I(w)}$ (spec.mx); for ${\mathrm{RH}}_{h}$: ${\hat{\mu }}_{MR}$ (rMh), ${\hat{\sigma^{2}}}_{MR}$ (rVh); for ${\mathrm{RH}}_{d}$: ${\hat{\mu }}_{MR}$ (rMd), ${\hat{\sigma^{2}}}_{MR}$ (rVd). Also, SSE (sse), kolg.t (kolg.t), $\hat{\sigma^{2}}$ (res.v), ω (omega), ${\hat{\alpha }}_{2}$ (pacf2), $S_{1}$ (s1), $S_{18}$ (s18), $S_{20}$ (s20), $S_{24}$ (s24), α (alpha), *v* (shape), ${\hat{\mu }}_{{\hat{\rho }}_{l}}$ (acf.m), ${\hat{Md}}_{{\hat{\rho }}_{l}}$ (acf.md).

An explanation about the most important variables, regardless of period and year, is presented below.

- M1: spec.mx, rMh, rMd, rVh, rVd, and pacf2 (see Table 1). The features rMh and rMd account for changes in the mean of the time series, while rVh and rVd are intended to explain changes in the variance. The rest of the features mentioned provide information about the dynamic structure of each time series. It was found that rMh, rMd, and rVh were important in the four periods considered, both in 2008 and 2010. rMd was relevant for WrA and WrB in 2008. The variable spec.mx was relevant in WrA and WrB for 2008 and 2010, as well as WrB. The variable pacf2 was found in WrB 2010. Hence, consistent results were derived from the two years under study.
- M2: sse, kolg.d, and spec.mx (computed from the residuals), as well as b, s1, s18, s19, s20, and s24 (from the models). From the residuals, sse accounts for the variance that is not explained by the models. This parameter appeared as important in all periods considered, except WrA 2010. kolg.d quantifies the deviation from normality for the residuals, and was relevant in all periods except Sp 2008 and WrB 2010. The third feature, spec.mx, which provides information about the dynamic structure of each time series, was relevant for all periods except WrB 2008, WrA 2010, and Sp 2010. Regarding the parameters computed from the models, b is related to the trend component of the time series, which was important in WrA 2010. The other variables mentioned are related to the seasonal components of the time series, which were shown to be important in Sm 2010.
- M3: res.v, shape, spec.mx, acf.m, and acf.md (computed from the residuals), as well as omega and alpha (from the model). From the residuals, res.v is aimed to explain the variance not explained by the models. It was relevant in all periods except Sp and Sm 2008. The variable shape provides information about the distribution of residuals, but it was only relevant in WrA 2010. The other features (i.e., spec.mx, acf.m, and acf.md) are intended to describe the dynamic structure of each time series. Spec.mx was important in all periods except Sp and Sm 2008, while the last two only appeared in Sp 2010. Regarding the parameters from the models, omega explains the changes in the mean of the conditional variance, while alpha quantifies the impact of the rotation on the conditional variance. The variable alpha only appeared in WrA 2010. Again, the fact that most variables were common in the three periods and in both years suggests strong consistency in the underlying phenomena explaining the discrimination between sensors.

In all cases, the classification variables corresponded to the different parts of the time series (WrA, WrB, Sp, and Sm), except for M3 in 2008 which only showed variables from winter (see Table 1).

**Figure 10:**
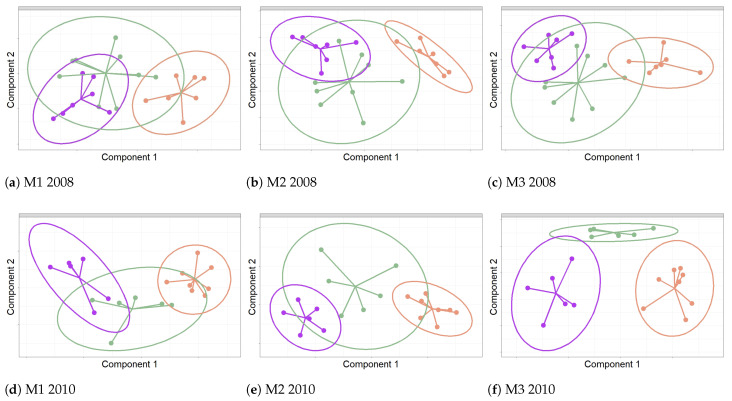
Discrimination of the time series of RH according to the position of sensors: Frescoes (F), Cornice and Ribs(RC), and Wall (W). Color codes: F sensors are shown in green, RC in orange, and W in purple. Graphics correspond to the projection of sensors over the first two components from sPLS-DA. Each graph shows confidence ellipses for each class to highlight the strength of the discrimination at a confidence level of 95%.

The results shown in Figure 10 correspond to the score plots for the first two components from sPLS-DA applied to the classification of sensors. They depict their projection over the two principal latent structures that best discriminate sensors according to their position. In 2008, the first component for each method allowed a rather good discrimination of sensors at the RC position with respect to the rest, though a poor discrimination was achieved between F and W (see Figure 10a–c).

In 2010, the first and second components for M3 displayed a clear discrimination between sensors located on the three positions. However, for M1 and M2, only RC sensors appear far apart from those on the walls, while the F group is located in between (see Figure 10d–f).

**Figure 11:**
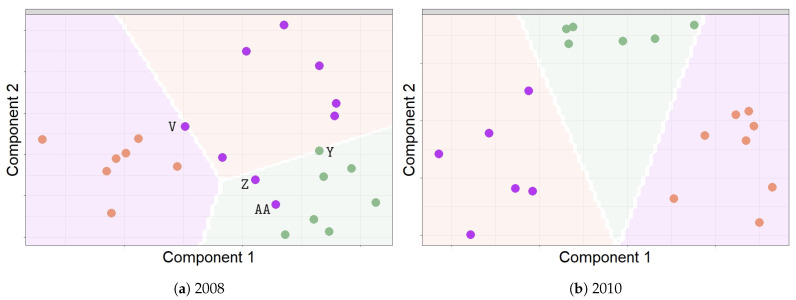
Prediction classes derived from M3: these plots display the predicted classes of each sensor located in different points in a simulated grid. The final classification prediction for the position of the sensors is displayed. The points in orange (RC), purple (W) and green (F) represent the class prediction for the sensors in the study. (**a**) For 2008: according to the classification prediction, the wrongly classified sensors were as follows: V, Y, Z, and AA. (**b**) For 2010: all sensors were classified correctly.

Regarding the performance of the three methods for achieving the classification of sensors, the best results were derived from M3 and the worst from M1. M3 yielded higher correct classification percentages: 77.40% in 2008 and 87.19% in 2010 (see Table 1b). For 2008, the final classification resulting from M3 variables displayed the following wrongly classified sensors: Y, AA, Z, and V (see Figure 11a). Three of them (Y, Z, and AA) were installed near the location where the salt efflorescence was found. For 2010, the final results from sPLS-DA for M3 showed that all sensors were classified correctly (see Figure 11b).

## 4. Discussion

The methodology proposed here consists of using sPLS-DA to classify time series of RH according to classification variables that were computed from different functions (e.g., sample ACF, sample PACF, spectral density, and MR). Additionally, the Seasonal ARIMA-TGARCH-Student model and the Additive SH-W method were used. Furthermore, estimated parameters of the models, as well as the mean, variance, and maximum values of the functions (e.g., sample ACF, sample PACF, spectral density, and statistics of the KS normality test, among others) were applied to the residuals derived from the models. The centroid distance was applied to classify the sensors, and the lasso penalty was used to select the optimal variables that determine the relevant components. Additionally, the BER parameter was employed to evaluate the performance of the classification methodology.

We used sPLS-DA because the classification data in this study are characterized by more variables than the number of time series (sensors), and in the interest of easily interpreting the results. This technique leads to underlying latent variables (components) that summarize the relevant information from the data for the purpose of discrimination. It performs variable selection for each component, which is an advantage. The key issue in time series clustering is how to characterize the similarities and dissimilarities between time series. Various metrics for measuring such similarity have been proposed, based on: parameters from models [83,84,85,86,87], serial features extracted from the original time series [88,89,90,91], the complexity of the time series [92,93,94,95,96,97], the properties of the predictions [98,99], and the comparison of raw data [100]. Regarding methods based on model parameters, the criterion most commonly considered is to assume that time series are properly explained by ARIMA processes. Piccolo [83] introduced the Euclidean distance between their corresponding AR expansion [38] as a metric and used a complete linkage clustering algorithm to construct a dendrogram. One problem of this metric is related to the numerical computations of AR coefficients. The same metric was also considered by Otranto [101] for dealing with GARCH processes. For ARMA models, Maharaj [102] developed an agglomerative hierarchical clustering procedure based on the *p*-value of a hypothesis test applied to every pair of stationary time series. Kalpakis et al. [103] studied the clustering of ARIMA time series by using the Euclidean distance between the linear predictive coding (LPC) cepstrum of two time series as their dissimilarity measure. Xiong and Yeung [104] classified univariate ARIMA time series by considering ARMA models. They derived an expectation maximization (EM) algorithm for estimating the coefficients and parameters of the models. However, if the underlying clusters are very close to each other, the clustering performance might diminish significantly. According to the review of the previously mentioned studies about the clustering of time series, it seems that the methodology applied here is rather unique because it uses a hybrid model comprising ARIMA and GARCH to calculate a distance for classifying time series. This is also probably the first using sPLS-DA in order to classify time series.

We found that the time series of RH, one per sensor, were very similar despite their different positions in the apse vault of the cathedral. When classifying the sensors, it turned out that few parameters appeared as relevant, most of which were features extracted from the residuals of models. This is most likely due to the similarity among the time series studied. As a consequence, the information that was not properly explained by the models was decisive for characterizing the differences between time series. The classification variables derived from the ARIMA-TGARCH-Student model yielded better performance than those from SH-W, which might suggest that the former model captures more information from the data than the latter. In fact, SH-W is an algorithm intended for producing point forecasts [60].

A comparison of the results from method M1 with those from the preliminary study of Zarzo et al. [1] indicates that a better classification was obtained here. The variables HMV and DMV (rMh and rMd) were relevant in both studies. Although the mean for the total observations was important in the preliminary project [1], this variable was not selected by the sPLS-DA. The classification variables selected per sPLS-DA explained the changes in the mean and variance of the time series with rMh, rMd, rVh, and rVd. Furthermore, the method obtains variables from sample PACF and spectral density which explain the autocorrelation of the time series. The research by Zarzo et al. [1] did not use variables related to the autocorrelation of the time series.

One disadvantage of sPLS-DA is the need to use the same number of classification variables for each sensor. As a consequence, a unique ARIMA-TGARCH-Student model was used for all sensors in the same part of the time series (WrA, WrB, Sp, and Sm). This means that a better fit might result, as it considers a different model for each time series. Another disadvantage is that it is necessary to know a priori the number of classes of the time series (sensors) for their classification. According to the previous idea, the limitations of the statistical methodology proposed in this study are: (1) sPLS-DA needs to know the number of classes before implementing the algorithm. (2) When applying a unique ARIMA-TGARCH parametric model to all sensors, it is unlikely that the best values for the classification variables will be found. This can affect the classification error rate of the sensors.

One advantage of using both sPLS-DA and ARIMA-TGARCH-Student is the capability of classifying time series with very similar characteristics. Additionally, the functions and models utilized here can be easily implemented because different packages are available in R software. One such example is the mixOmics package of R, which has different functions that allow sPLS-DA to be implemented simply and makes it easy to display the different results for interpretation. Furthermore, this package can handle missing values using the NIPALS approach. It takes advantage of the PLS algorithm which performs local regressions on the latent components. There are two main advantages of using PLS—it both handles missing values and calculates the components sequentially. In this study, the anomalous values of classification variables were considered as missing values in order to avoid possible problems with the classification of the sensors. The percentage of values that were used as missing were lower than 2%.

In relation to future studies, alternative classification variables could be considered depending on the different scenarios and according to the characteristics of the time series. In order to obtain classification variables that capture more information from the data, flexible models can be proposed. Some options for calculating the classification variables might be the following:

- Cepstral coefficients: Ioannou et al. [105] studied several clustering techniques in the context of the semiparametric model: spectral density ratio. They found that the cepstral- based techniques performed better than all the other spectral-domain-based methods, even for relatively small subsequences.
- Structural time series model: the flexibility required from this model can be achieved by letting the regression coefficients change over time [106].
- A nonparametric approach of the GARCH [107,108].

Regarding classification techniques when there are fewer variables than time series, sPLS-DA can be extended by using the elastic net [109] as the penalization. Finally, a further study might be carried out in controlled scenarios, where time series can be computationally simulated by controlling different characteristics in order to identify the strengths and weaknesses of the proposed methodology. In alignment with the previous ideas for improving the methodology, future research will use sPLS-DA with two methods: a nonparametric Seasonal ARIMA-GARCH model and a structural time series model. Furthermore, several time series will be computationally simulated in controlled scenarios in order to evaluate the results when using sPLS-DA, together with one of the previously mentioned methods.

García-Diego and Zarzo [2] concluded that the environment surrounding the Renaissance frescoes was not the same at all points of the apse vault of the cathedral. Sensors located on the walls or on the paintings registered higher RH values than those in the vault ribs. Thus, the mean value of RH is related with the three previously mentioned classes. The ideal goal is obviously to achieve a correct classification of all sensors. However, a poor classification error rate might be caused by either the malfunctioning of some sensors or a poor performance of the classification technique, or if there is a problem related with the microclimate where the sensors are located. Those sensors incorrectly classified by the technique should be checked to identify possible moisture problems in the artworks. In this work, the main cause of sensor malfunction was the development of salt deposits around the probes as a consequence of fitting some of the probes inside the layer of plaster supporting the frescoes.

The results indicate that sPLS-DA could be implemented for the online monitoring of fresco paintings aimed at preventive conservation using the parameters and features previously extracted from the hybrid models based on GARCH and ARIMA as classification variables. This analysis might be carried out for each season of every year.

## 5. Conclusions

The methodology proposed here is useful for understanding the differences in thermo-hygrometric conditions monitored inside large buildings or museums, which might provide a basis for better assessing the potential risks related to temperature and humidity on the artworks. Among the methods proposed, a hybrid approach based on ARIMA and GARCH models with sPLS-DA yielded the best performance. Parsimonious models with a small subset of components and classification variables were obtained using sPLS-DA, which offers satisfactory results with easy interpretation. Another advantage of sPLS-DA is that it can be implemented easily with mixOmics, which allows a focus on graphical representation in order to better understand the relationships between the different observations and variables. Furthermore, this package can deal with missing values. Finally, the use of a hybrid approach based on ARIMA and GARCH models as well as sPLS-DA is a novel proposal for classifying different time series. In order to improve the methodology proposed in this research, future research will use sPLS-DA with two methods that are more flexible than those applied in this study. This will capture more information from the data. Furthermore, a computational simulation will be carried out in order to evaluate the new methodology in different possible scenarios.

## Figures and Tables

**Table 1:** Results from sPLS-DA (2008 and 2010): variables selected for the first component per method (M) for each period (WrA stands for winter-A, WrB for winter-B, Sp for spring, and Sm for summer). With respect to the names of the selected classification variables, a prefix is used to indicate the period of the time series that was used to apply the three methods. For example, WrA was used as a prefix in the name of the classification variables to indicate the part of the time series where the variables came from. Thus, $WrA_{rMh}$ indicates that the variable rMh corresponds to period WrA. For each component, variables are ordered according to the absolute value of their loading weights, from highest to lowest. Variables with negative weights are highlighted in bold. The balanced classification error rate (BER) is indicated for the first component per method.

(**a**) Results from sPLS-DA (2008).		
**Method**	**Variables**	**BER**
M1	${\mathrm{WrA}}_{\mathrm{spec}.\mathrm{mx}}$, ${\mathrm{WrA}}_{\mathrm{rMh}}$, ${\mathrm{WrB}}_{\mathrm{rMh}}$, ${\mathrm{Sp}}_{\mathrm{rMh}}$, ${\mathrm{Sm}}_{\mathrm{rMh}}$, ${\mathrm{WrA}}_{\mathrm{rVh}}$, ${\mathrm{WrB}}_{\mathrm{rVh}}$	30.02%
	${\mathrm{Sp}}_{\mathrm{rVh}}$, ${\mathrm{Sm}}_{\mathrm{rVh}}$, ${\mathrm{WrA}}_{\mathrm{rMd}}$, ${\mathrm{WrB}}_{\mathrm{rMd}}$, ${\mathrm{Sp}}_{\mathrm{rMd}}$, ${\mathrm{Sm}}_{\mathrm{rMd}}$, ${\mathrm{WrA}}_{\mathrm{rVd}}$, ${\mathrm{WrB}}_{\mathrm{rVd}}$	
M2	$WrA_{sse}$, $WrA_{spec.mx}$, $WrB_{sse}$, $Sp_{sse}$, ${\mathrm{WrA}}_{\mathrm{kolg}.t}$, $Sp_{spec.mx}$, ${\mathrm{WrB}}_{\mathrm{kolg}.t}$	24.05%
	${\mathrm{Sm}}_{\mathrm{kolg}.t}$, $Sm_{sse}$, $Sm_{spec.mx}$	
M3	${\mathrm{WrA}}_{\mathrm{spec}.\mathrm{mx}}$, ${\mathrm{WrA}}_{\mathrm{res}.v}$, ${\mathrm{WrB}}_{\mathrm{spec}.\mathrm{mx}}$, ${\mathrm{WrB}}_{\mathrm{res}.v}$, ${\mathrm{WrA}}_{\mathrm{omega}}$	22.60%
(**b**) Results from sPLS-DA (2010).		
**Method**	**Variables**	**BER**
M1	$WrA_{spec.mx}$, $WrA_{rMd}$, $WrB_{rMd}$, $Sp_{rMd}$, $Sm_{rMd}$, $WrA_{rMh}$, $WrB_{rMh}$	24.08%
	$Sp_{rMh}$, $Sm_{rMh}$, $WrA_{rVh}$, $WrB_{rVh}$, $Sp_{rVh}$, $Sm_{rVh}$, $WrB_{spec.mx}$, ${\mathrm{WrB}}_{\mathrm{pacf}2}$	
M2	$WrB_{spec.mx}$, $Sm_{sse}$, ${\mathrm{Sp}}_{\mathrm{kolg}.t}$, $Sp_{sse}$, ${\mathrm{WrA}}_{\mathrm{kolg}.t}$, $WrB_{s1}$, ${\mathrm{Sm}}_{\mathrm{kolg}.t}$, $WrB_{sse}$	21.17%
	$WrB_{s24}$, $Sm_{spec.mx}$, ${\mathrm{Sm}}_{s19}$, ${\mathrm{Sm}}_{s18}$, ${\mathrm{WrA}}_{b}$, ${\mathrm{Sm}}_{s20}$, $Sp_{s24}$	
M3	$Sp_{res.v}$, $Sm_{res.v}$, $Sm_{spec.mx}$, $WrA_{res.v}$, $WrB_{res.v}$, $Sp_{spec.mx}$, $Sp_{omega}$	12.81%
	$Sm_{omega}$, $WrA_{spec.mx}$, $WrB_{omega}$, ${\mathrm{WrB}}_{\mathrm{spec}.\mathrm{mx}}$, ${\mathrm{WrA}}_{\mathrm{alpha}}$, ${\mathrm{WrA}}_{\mathrm{shape}}$${\mathrm{Sp}}_{\mathrm{acf}.\mathrm{md}}$${\mathrm{Sp}}_{\mathrm{acf}.m}$	

## Data Availability

The data sets used are available at: https://zenodo.org/record/4412126 DOI: 10.5281/zenodo.4412126.
